# Management of Microbiological Contamination of the Water Network of a Newly Built Hospital Pavilion

**DOI:** 10.3390/pathogens10010075

**Published:** 2021-01-16

**Authors:** Osvalda De Giglio, Giusy Diella, Marco Lopuzzo, Francesco Triggiano, Carla Calia, Chrysovalentinos Pousis, Fabrizio Fasano, Giuseppe Calabrese, Vincenza Rafaschieri, Lucia Federica Carpagnano, Matilde Carlucci, Loreto Gesualdo, Maria Luisa Ricci, Maria Scaturro, Maria Cristina Rota, Lucia Bonadonna, Luca Lucentini, Maria Teresa Montagna

**Affiliations:** 1 Regional Reference Laboratory of Clinical and Environmental Surveillance of Legionellosis, Department of Biomedical Science and Human Oncology, University of Bari Aldo Moro, Piazza G. Cesare 11, 70124 Bari, Italy; osvalda.degiglio@uniba.it (O.D.G.); giusy.diella@uniba.it (G.D.); marcolopuzzo@gmail.com (M.L.); francesco.triggiano@uniba.it (F.T.); carla.calia@uniba.it (C.C.); vpousis@gmail.com (C.P.); fabrizio.fasano1979@libero.it (F.F.); 2 A.O.U. Policlinico di Bari, 70124 Bari, Italy; giuseppe.calabrese@policlinico.ba.it (G.C.); vincenza.rafaschieri@policlinico.ba.it (V.R.); dr.fedecarpagnano@gmail.com (L.F.C.); matilde.carlucci@policlinico.ba.it (M.C.); 3 Department of Emergency and Organ Transplantation-Nephrology, Dialysis and Transplantation Unit, University of Bari Aldo Moro, 70124 Bari, Italy; loreto.gesualdo@uniba.it; 4 Department of Infectious Diseases, Istituto Superiore di Sanità, Viale Regina Elena, 00161 Rome, Italy; marialuisa.ricci@iss.it (M.L.R.); maria.scaturro@iss.it (M.S.); mariacristina.rota@iss.it (M.C.R.); 5 Department of Environment and Health, Istituto Superiore di Sanità, Viale Regina Elena, 00161 Rome, Italy; lucia.bonadonna@iss.it (L.B.); luca.lucentini@iss.it (L.L.)

**Keywords:** *Legionella*, legionellosis, water networks, *P. aeruginosa*, *E. coli*, enterococci, coliforms, waterborne diseases, new pavilion, hospital

## Abstract

The good installation, as well as commissioning plan, of a water network is a crucial step in reducing the risk of waterborne diseases. The aim of this study was to monitor the microbiological quality of water from a newly built pavilion before it commenced operation. Overall, 91 water samples were tested for coliforms, *Escherichia coli*, enterococci, *Pseudomonas aeruginosa* and *Legionella* at three different times: T0 (without any water treatment), T1 (after treatment with hydrogen peroxide and silver ions at initial concentration of 20 mg/L and after flushing of water for 20 min/day for seven successive days) and T2 (15 days later). Coliforms were detected in 47.3% of samples at T0, 36.3% at T1 and 4.4% at T2. *E. coli* was isolated in 4.4% of the samples only at T1, while enterococci appeared in 12.1% of the samples at T1 and in 2.2% at T2. *P. aeruginosa* was isolated in 50.5% of the samples at T0, 29.7% at T1 and 1.1% at T2. *Legionella pneumophila* serogroup 8 was isolated in 80.2% of the samples at T0, 36.3% at T1 and 2.2% at T2. Our results confirmed the need for a water safety plan in new hospital pavilions to prevent the risk of waterborne diseases.

## 1. Introduction

In health facilities, the water used for drinking, hygiene and medical purposes can affect the health of patients, staff and other users of the facility, as the quality standards prescribed by national regulations controlling the quality of drinking water, the Italian Legislative Decree no. 31 of 2001 (Lgs.D.31/01) [1], are not always able to guarantee the safety of vulnerable patients [2]. Some hydrophilic microorganisms (e.g., *Pseudomonas aeruginosa*, *Legionella*, fungi) can cause serious infections if present in the water used to wash wounds, burns, medical devices and humidifiers, with the risk of an inauspicious outcome in patients with a high risk of infection [2,3,4,5,6,7,8]. 

Enteric pathogens of fecal origin can enter the water supply due to accidental malfunction of the sewage system, while environmental organisms, such as *Legionella*, *Pseudomonas* and fungi, can grow in systems that use water (e.g., cooling towers) or in water networks in which the flow is not continuous [9]. Even new buildings and/or hospital renovations can cause malfunctions of the plumbing system as vibrations or significant changes in the water pressure can lead to the detachment of biofilms, releasing microorganisms into the water. Furthermore, soil could enter the system during the construction of new network sections [2]. 

In recent years, particular attention has been paid to nosocomial legionellosis [8,10,11,12,13,14]. The association between drinking water and nosocomial legionellosis was first described about 40 years ago [15]. The complexity of hospital water systems and the vulnerability of hospitalized patients increase the risk of *Legionella* transmission with severe outcomes. The new European Drinking Water Directive introduces a new approach that includes the assessment of possible risks, including legionellosis, deriving from domestic distribution systems [16]. Various disinfection techniques (chemical disinfection, ultraviolet light and high temperature) were employed as water disinfection systems. Most of them were found to be useful to control the average load of *Legionella*, but its complete eradication has not yet been demonstrated [17].

*Legionella* replicates between 20 °C and 50 °C, with more rapid growth occurring when temperatures approach 40 °C [18]. Whereas most hydrophilic bacteria do not survive such high temperatures, *Legionella* adapts easily by continuing to reproduce within amoebae and can cause a severe form of pneumonia known as Legionnaires’ disease [8,11,19,20]. In Italy, 2964 cases of legionellosis were reported in 2018 (incidence rate = 48.9 cases per 1 million inhabitants) of which 3.4% were of nosocomial origin [21].

The aim of this study was to manage and monitor the microbiological quality of the water network in a newly built hospital pavilion located in a large university hospital in Apulia (southern Italy), before it commenced operation. 

## 2. Results

### 2.1. Physical and Chemical Parameters of Water Sample

Table 1 shows the mean value of results of the physico-chemical analysis carried out on the water samples collected at time T0 (without any water treatment), T1 (after treatment with hydrogen peroxide and silver ions at initial concentration of 20 mg/L and after flushing water for 20 min/day for seven successive days) and T2 (15 days later). The limit values recommended by Lgs.D. 31/01 were reported.

### 2.2. Bacterial Detection

**Figure 1:**
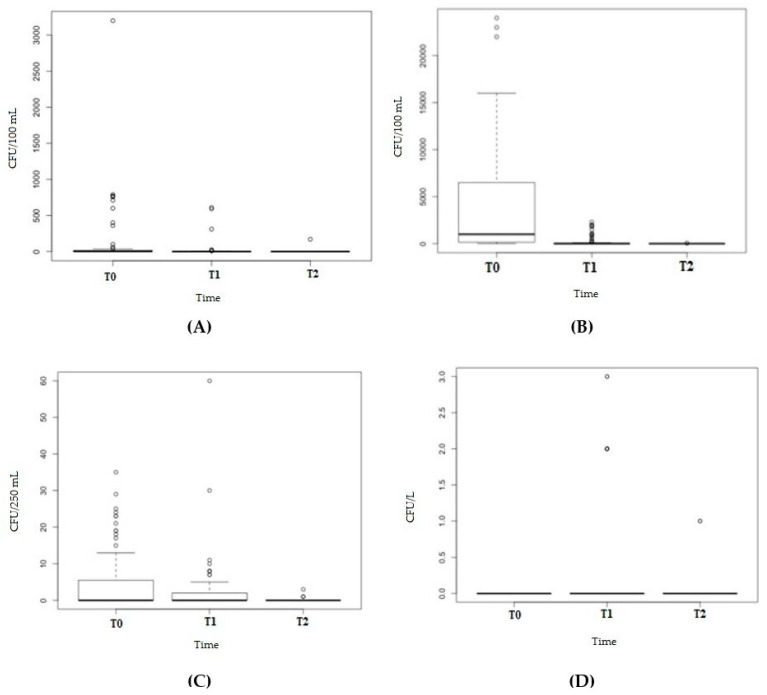
Box plot of the coliform load (CFU/100 mL) (**A**), enterococci load (CFU/100 mL) (**B**), *P. aeruginosa* load (CFU/250 mL) (**C**) and *L. pneumophila* load (CFU/L) (**D**), at T0, T1 and T2; CFU = colony forming units; ° = outlier values.

Coliforms were detected in 47.3% (43/91) of water samples at T0, 36.3% (33/91) at T1 and 4.4% (4/91) at T2. A statistically significant difference was detected at T2 compared with T0 (χ^2^ Yates correction = 41.4197, *p*-value < 0.0001) and T1 (χ^2^ Yates correction = 26.5961, *p*-value < 0.0001). The Lilliefors (Kolmogorov–Smirnov) normality test showed no normal distribution of the load (D = 0.26741, *p*-value < 0.0001). The median value of the coliform load was <1 CFU/100 mL at T0 (range < 1–35), T1 (range < 1–60) and T2 (range < 1–3) (Figure 1A). The Friedman rank-sum test (χ^2^ = 50.429, df = 2, *p*-value < 0.0001) and post-hoc Conover’s test for a two-way analysis (each analysis with *p*-value < 0.001) showed statistically significant differences in coliform load at T0, T1 and T2. There were no statistically significant differences in the positivity rate (Table 2) and load (Table 3) of coliforms between taps and showers.

*Escherichia coli* was detected in 4.4% (4/91) of samples only at T1, with no statistically significant differences in the positivity rate over time (Fisher’s F test *p*-value = 0.12) (Table 2). The median value of the load was <1 CFU/100 mL (range < 1–2) (Table 3). 

Enterococci (Figure 1B) were not isolated at T0, but they appeared in 12.1% (11/91) of samples at T1 (median load value = <1 CFU/100 mL, range < 1–3) and in 2.2% (2/91) at T2 (each, 1 CFU/100 mL). Statistically significant differences were present among the positivity rates between T0 and T1 (0% vs. 12.1% Fisher’s F test *p*-value = 0.0007), between T1 and T2 (12.1% vs. 2.2% Fisher’s F test *p*-value = 0.02), but not between T0 and T2 (0% vs. 2.2%, Fisher’s F test *p*-value = 0.49). The Lilliefors (Kolmogorov–Smirnov) normality test showed no normal distribution of the load (D = 0.53728, *p*-value < 0.0001). The Friedman rank-sum test (Friedman χ^2^= 18.216, df = 2, *p*-value = 0.0001) and post-hoc Conover’s test showed statistically significant differences (*p*-value < 0.001) in the enterococci load at T0, T1 and T2. 

No statistically significant differences in the enterococci positivity rate between taps and showers were detected at the different time points (Table 2). Showers showed a higher enterococci load than taps at T2 (W = 737, *p*-value = 0.02) (Table 3). 

The *P. aeruginosa* positivity rate showed a decrease at different times: from 50.5% at T0 to 29.7% at T1 (T0 vs. T1: χ^2^ Yates correction = 7.4108, *p*-value = 0.006) and to 1.1% at T2 (both T0 vs. T1 and T1 vs. T2: Fisher’s F test *p*-value < 0.0001). The Lilliefors (Kolmogorov–Smirnov) normality test showed no normal distribution of the load (D = 0.43874, *p*-value < 0.0001). The median value of *P. aeruginosa* was <1 CFU/250 mL at T0 (range < 1–3200), at T1 (range < 1–610) and at T2 (range < 1–170) (Figure 1C). The Friedman rank-sum test (Friedman χ^2^ = 47.041, df = 2, *p*-value < 0.0001) and post-hoc Conover’s test showed statistically significant differences (*p*-value < 0.0001) in the *P. aeruginosa* load at different time periods (T0, T1 and T2), revealing a decrease in the load. There were no statistically significant differences between taps and showers in terms of the positivity rate (Table 2) and load (Table 3) of *P. aeruginosa*. 

### 2.3. Legionella Detection

*Legionella pneumophila* sg 8 was isolated from 80.2% (73/91) of water samples at T0, from 36.3% (33/91) at T1 (T0 vs. T1—χ^2^ Yates correction = 34.36; *p*-value < 0.00001) and from 2.2% (2/91) at T2 (both T0 vs. T2 and T1 vs. T2 Fisher’s F *p*-value < 0.0001). The Lilliefors (Kolmogorov–Smirnov) normality test showed no normal distribution of the load (D = 0.24695, *p*-value < 0.0001). The median value of the load resulted in 1000 CFU/L (range < 50–24,000) at T0 and <50 CFU/L (range < 50–2300) at T1 and T2 (range <50–50) (Figure 1D). The difference between *L. pneumophila* loads at different time periods during the analysis was statistically significant (Friedman χ^2^ = 112.87, df = 2, *p* < 0.0001—post hoc Conover’s test for a two-way analysis with *p*-value < 0.0001). 

Table 2 shows a comparison of the *L. pneumophila* positivity rates between taps and showers, with a statistically significant difference only detected at T2 (Fisher’s F-test *p*-value = 0–0.07% vs. 8.3%). Taps showed a higher load than showers at T0 (Wilcoxon rank-sum test with continuity correction W = 1091.5, *p*-value = 0.01) and a lower load at T2 (W = 737, *p*-value = 0.02) (Table 3).

A Poisson regression model was used to perform multivariate analysis of the *L. pneumophila* load compared with the load of other microorganisms (Table 4).

The results showed a large increase in the relative risk (RR) of the *L. pneumophila* load for each increase in the coliform load (+1.449%). The *P. aeruginosa* load was directly proportional to the *L. pneumophila* load, whereas the *E. coli* and enterococci loads were inversely proportional to the *L. pneumophila* load.

## 3. Discussion

The water safety in healthcare facilities could be underestimated in a new building. According to Guidelines for the Design and Construction of Health Care Facilities [22], a new construction must follow a control plan for the water network, including *Legionella* investigation [23]. This plan should contain a risk assessment that identifies water treatment systems and points of water use that require intervention control strategies to mitigate potential hazards. The installation, modification and maintenance of plumbing systems must be adequate, by virtue of the awareness of the overall scheme of the system and its operation [2]. To achieve this goal, a multidisciplinary approach is necessary and should involve epidemiologists, hospital safety officers, architects, engineers, as well as clinicians and microbiologists. 

According to previous studies [9,12], our results revealed high microbiological contamination of the water network, probably due to the long period of inactivity before the inauguration of the new pavilion. While Gram-negative bacteria and fungi tend to adhere to biofilms at or near distal points of use [3,8,24], *Legionella* can also colonize deep hospital infrastructures and is responsible for nosocomial cases of legionellosis [10,25]. Fortunately, as reported by some Authors [17], our remediation interventions (i.e., flushing with hydrogen peroxide and silver ions for 20 min/day for one week) resulted in an overall decrease in the bacterial load. 

According to some Authors, the protocol undertaken showed an important role in preventing biofilm formation, which can support Legionella growth [26,27]. We wanted to examine both the taps and the showers to see if there were different positivity rates and microbial loads. Our results showed that all remediation interventions were less effective for *Legionella* between T0 and T2 for showers than for taps. According to a previous report [28], shower hoses promote bacterial growth near a critical end-user exposure path within the building’s drinking water pipes. They are typically exposed to warm water (rather than just cold or hot) and would be subject to distal end cooling, even when used with properly regulated hot water recirculation systems [29]. 

The hydrogen peroxide and silver ions are active agents against bacteria, yeast, fungi, viruses, spores, proto-, and metazoans [30]; they are included in the Italian Guidelines for the Control and Prevention of Legionellosis [31]. It is compatible with different pipeline materials and does not react with the organic constituents in the water to form dangerous residues with respect to chlorine, sodium hypochlorite and monochloramine treatment [32]. European directives do not establish a concentration limit for hydrogen peroxide in drinking water, although the German and British version of the EN 902:2016 [33] provides a dosage up to 17 mg/L [17]. The hydrogen peroxide and silver ions are stable at high temperatures, and its disinfection power increases significantly as water temperature increases [31].

According to some studies, our results confirmed that the water distribution system consisting of galvanized iron, as opposed to plastic material (polyethylene and polyvinyl chloride) [34,35], together with prolonged use of the disinfectant, inhibited the colonization of Legionella [31].

Regarding the practice of flushing as preventive measure, although this information is specific to Legionella species, our study showed that it may also provide a benefit in reducing the concentration of other waterborne pathogens and potential exposure [36]. 

Waterborne Gram-negative bacteria (other than *Legionella*) that may infect hospitalized patients may be introduced into the water supply via colonized patients, and then spread through the environment [23]. Although the role of the environment is unclear in many nosocomial outbreaks, sink drains have been repeatedly implicated in reports of the transmission of Gram-negative bacteria, including *Pseudomonas* and *Klebsiella* [36,37]. The attribution of an outbreak to sink drain contamination was usually based on similar genotypic patterns and the termination of the outbreak following remediation of the contaminated sink drain [37,38,39].

In conclusion, poor water system design in new hospital buildings or limited use of the water network, if not properly managed, can represent a danger for patients and healthcare professionals, as evidenced by some recorded outbreaks (due to *Legionella* or *P. aeruginosa*) in newly built hospitals in Germany. In one of these outbreaks, *P. aeruginosa* colonization was not eliminated even after heat treatment and continued disinfection with chlorine dioxide. As a result, the building was vacated [9]. Therefore, the Water Safety Plan approach is required to focus on managing risks throughout all steps in the water supply chain from source water catchment through treatment processes to storage, distribution and handling of drinking water [40]. In new or renovated buildings, adequate planning of microbiological checks of the water network before the start of activities is the first step in minimizing infectious risks. Malfunction of the water network and the corrective actions to reestablish the water quality (e.g., emptying the system, especially if it is not possible to maintain weekly flushing), can be very expensive and can include the transfer of patients and/or the suspension of activities within a ward. Furthermore, if the level of microbial contamination, as in the case of *Legionella*, varies over a short period of time [41,42], inadequate sampling can lead to ill-informed decision-making (e.g., whether or not to disinfect, or what type of treatment to adopt), compromising the safe and effective management of hospital wards.

## 4. Materials and Methods 

### 4.1. Study Design 

This study was carried out from April to May, 2020, in a large university hospital in Apulia, southern Italy, which comprised 1400 beds in 33 separate buildings. A new seven-story building (basement, ground floor and five floors) covering a total of 6000 square meters was selected for this study. The installation of the water system powered by municipal water and equipped with galvanized iron pipelines was started in 2018 and ended in February 2020. In April 2020, within the Water Safety Plan (WSP) implementation program, a systematic monitoring and disinfection program of water network was started.

Water samples from all of the end points of use (67 taps and 24 showers—91 samples in total) were collected in April 2020 (T0, without any water treatment), for a microbiological check before the inauguration. The following month (T1, i.e., after disinfection of the water network with hydrogen peroxide and silver ions and after flushing water for 20 min every day for seven successive days) and 15 days later (T2) the sampling was repeated. 

Physico-chemical and microbiological parameters (coliforms, *E. coli*, enterococci, *P. aeruginosa*) and *Legionella* were investigated using the methods described below. 

### 4.2. Water Disinfection

The method of disinfection with hydrogen peroxide and silver ions was continuous, i.e., without interrupting the water to the user. The injection of product was performed at make-up cold water pipe to the hot water boiler and was normally equal to 20 mg/L. A pulse-emitting counter was installed on the make-up pipe to the boiler in order to control the dosage of the product in proportion to the flow of water supplied to the user. Once the dosage was calibrated, it was advisable to periodically check the concentration of residual product at the users and in the recirculation pipe, which must remain between 10 and 15 mg /L of product detected with the control kit (Cillichemie, Milan, Italy). 

Together with disinfectant, to prevent the formation of any limescale deposits or the initiation of corrosion processes, an anticorrosive product based on sodium hydroxide (2.5–10%), sodium carbonate (≤ 2.5%), salicylic acid, sodium salt (≤ 2.5%) and phosphoric acid (≤ 2.5%) was administered continuously.

### 4.3. Physico-Chemical Parameters

Each sample was analyzed for the physico-chemical parameters provided by Lgs. D. 31/01 [1]. The temperature, pH and conductivity were measured with a multiparametric probe (Edge, Hanna Instrument Inc.; Woonsocket, RI, USA). Chemical parameters (hardness, free chlorine, chlorides, ammonium, nitrites, nitrates) were analyzed by colorimetric reaction with Spectrophotometer HI83399 (Hanna Instrument Inc; Woonsocket, RI, USA).

In particular, the measurement of ammonia concentration in the solution was based on the ASTM Manual of Water and Environmental Technology, D1426-92, Nessler method [43], (HI 93733 reagent). Free chlorine was evaluated following the US EPA (United States Environmental Protection Agency) Method 330.5 [43] with N, N-diethyl-p-phenylene diamine (DPD, HI 93701 reagent). The EPA Diazotization method 354.1 [43] was used to analyze Nitrite (HI 93707 reagent). Total hardness was evaluated using the EPA recommended method 130.1 (HI 93735 reagent) [44]. The Cadmium Reduction Method (Method EPA 353.2) was used for the colorimetric determination of nitrate as nitrogen (HI 93728 reagent) [44]. The Mercury (II) Thiocyanate Method (Method EPA 325.2) with the specific reagent (HI 93753 reagent) was used for the colorimetric determination of chloride [44].

### 4.4. Microbiological Investigation

Sampling and processing procedures were performed according to the Italian Lgs.D.31/01 [1] relating to coliforms, *Escherichia coli*, enterococci and *Pseudomonas aeruginosa*. Cold water samples (1 L) were collected from all taps and showers in sterile bottles with sodium thiosulphate pentahydrate (0.01%, *w*/*v*), to neutralize the chlorine present in water samples, and transported to the laboratory at 4 °C to be analyzed within 4 h. Specific aliquots of each sample were filtered through a cellulose ester membrane with a diameter of 47 mm and a pore size of 0.45 µm (Millipore, Milan, Italy).

For *E. coli* and coliform investigations, 100 mL of each water sample was filtered, and the membrane placed on plates containing Chromogenic Coliform Agar (Biolife Italiana Srl, Milan, Italy). After incubation at 36 ± 2 °C for 24 ± 2 h, the blue-violet colonies were identified as *E. coli*, and the salmon pink, oxidase-negative colonies were identified as coliforms [45]. 

For the isolation of enterococci, 100 mL of the sample was filtered; the membrane was placed on Slanetz and Bartley agar medium (Biolife Italiana Srl, Milan, Italy) and incubated at 36 ± 1 °C for 48 h. When dark pink-red colonies developed the membrane was transferred to a plate containing Bile Esculin Azide agar (Biolife Italiana Srl, Milan, Italy) and incubated at 44 °C for 2 h. Brown colonies with brown-black halos and positive catalysis were identified as enterococci [46].

*P. aeruginosa* was investigated in 250 mL of sample. After sample filtration, the membrane was placed on a plate containing Pseudomonas Selective Agar supplemented with cetrimide (0.20 g) and nalidixic acid (15 mg) (Microbiol, Cagliari, Italy) and incubated at 36 ± 2 °C for 44 ± 4 h. Blue-green pyocyanin-producing colonies were directly confirmed to be *P. aeruginosa* [47].

The samples were considered compliant to Lgs.D.31/01 when *E. coli*, coliforms and enterococci were absent from 100 mL of each sample (limit of detection, LOD < 1 CFU/100 mL), and *P. aeruginosa* was absent from 250 mL of sample (LOD < 1 CFU/250 mL).

### 4.5. Legionella Investigation

Hot water samples (1L) were collected from all showers and taps, in sterile dark glass containers containing sodium thiosulphate pentahydrate (0.01%, *w*/*v*) to neutralize chlorine present in the water and were transported immediately at environmental temperature (19.1 °C; range 18.7–24.1 °C) to be analyzed within 24 h [30]. Each sample was filtered through a 0.2 μm isopore nylon membrane, 47 mm in diameter (Millipore Corporation, Bedford, MA, USA). Each membrane was suspended in 10 mL of the same water sample and vortexed. After, 200 μL of each sample was seeded onto GVPC (glycine vancomycin polymyxin cycloheximide) agar plates (Liofilchem Srl, Teramo, Italy), incubated at 37 ± 1 °C for 10 days in a humid environment (under 2.5% CO_2_) and examined after 2, 4 and 10 d of incubation. Suspect colonies were subcultured on buffered charcoal yeast extract (BCYE) agar (BioMérieux, Marcy-l’Etoile, France) with and without L-cysteine. Colonies that grew only in the presence of cysteine were identified as *Legionella* and confirmed using a latex agglutination test with polyvalent (Biolife Italiana Srl, Milan, Italy) and monovalent antisera (Biogenetics Srl, Tokyo, Japan). Water samples containing < 50 colony-forming units per liter (CFU/L) were considered negative for *Legionella* [48]; this concentration falls within the threshold below which no intervention is required in healthcare facilities [31].

### 4.6. Statistical Analysis

The Lilliefors (Kolmogorov–Smirnov) normality test was used to verify the normality of the distribution of the differences between the microbiological parameter values and the *Legionella* load at the T0 (before water treatment) and T2 time periods (after the second water treatment) [49]. 

The Friedman rank sum test and post-hoc Conover’s test for two-way analysis or the Wilcoxon signed rank test with continuity correction was used to compare the values of the potable parameters and *Legionella* loads in different analysis periods (paired data) or between taps and showers.

Either Fisher’s exact test or the χ^2^ test with Yates correction was used to compare the positivity of the potable parameters in the difference time period or between taps and showers. A Poisson regression model was used to perform multivariate analysis of the *Legionella* load compared with the loads of other microbiological parameters.

The final model included only variables with a p-value of < 0.05 in the preliminary model of all variables. To quantify the effects of the microbiological parameter load (other than *Legionella*), we computed the influences (eβ-1) that corresponded to the relative risk (RR) [19]. R software version 3.5.1 was used for statistical analysis, and a *p*-value < 0.05 was considered statistically significant.

## 5. Conclusions

Different bacteria may contaminate nosocomial water systems, therefore, an active and well-planned environmental surveillance strategy in hospitals is vital for prevention. In particular, public health agencies should assess the “Water Safety Plans” for new buildings and new or renovated water systems where health risks can be high. 

Based on our findings, we advise that, before occupying a new pavilion, accurate environmental microbiological surveillance should be performed to reduce the risk of waterborne diseases.

## Figures and Tables

**Table 1:** Physical and chemical parameters of water from sampling the end points of use.

Parameters	Cold Water		Hot Water		Limit Value ^1^
	Value		Value		
	Mean	Range	Mean	Range	
pH	8.13	7.9–9.0	8.16	7.9–9.0	≥6.5 to ≤9.5
T (°C)	15.8	13.4–16.3	56.5	50.1–57.2	-
Conductivity (µS/cm)	650	640–685	647	640–680	2500
Hardness (°F)	16.2	16–18	15.3	15–17	15–50
Free Chlorine (mg/L)	0.01	0.00–0.01	0.01	0.00–0.01	0.2
Chlorides (mg/L)	30	26–32	28	25–30	250
Ammonium (mg/L)	<0.10	-	<0.10	-	0.50
Nitrites (mg/L)	<0.10	-	<0.10	-	0.50
Nitrates (mg/L)	0.7	0.6–1.2	0.6	0.5–1.0	50
Disinfectant residual (mg/L)	21.0	20.0–28.0	23.7	20.0–29.7	-
Flow rate (L/h)	550	450–650	550	450–650	-

^1^ According to the Italian Legislative Decree no. 31 of 2001 (Lgs.D. 31/01). - Not available.

**Table 2:** Comparison of the positivity rates between taps and showers.

Microorganism	Period	Tap (No./No.)	Shower (No./No.)	Test
Coliforms	T0	46.3 (31/67)	50.0 (12/24)	χ2 = 0.0058, *p*-value = 0.94
	T1	35.8 (24/67)	37.5 (9/24)	χ2 = 0.0101, *p*-value = 0.92
	T2	4.5 (3/67)	4.2 (1/24)	Fisher’s F test *p*-value = 1
	T0	0 (0/67)	0 (0/24)	Fisher’s F test *p*-value = 1
*E. coli*	T1	4.4 (4/67)	0 (0/24)	Fisher’s F test *p*-value = 0.57
	T2	0 (0/67)	0 (0/24)	Fisher’s F test *p*-value = 1
Enterococci	T0	0% (0/67)	0% (0/24)	Fisher’s F test *p*-value = 1
	T1	13.4% (9/67)	8.3% (2/24)	Fisher’s F test *p*-value = 0.72
	T2	0% (0/67)	8.3% (2/24)	Fisher’s F test *p*-value = 0.07
*P. aeruginosa*	T0	46.3% (31/67)	62.5% (15/24)	χ2 = 1.2696, *p*-value = 0.26
	T1	29.9% (20/67)	29.2% (7/24)	χ2 = 0.039, *p*-value = 0.84
	T2	0% (0/67)	4.2% (1/24)	Fisher’s F test *p*-value = 0.27
*L. pneumophila*	T0	85.1% (57/67)	66.7% (16/24)	χ2 = 2.70, *p*-value = 0.10
	T1	37.3% (25/67)	33.3% (8/24)	χ2 = 0.01, *p*-value = 0.91
	T2	0% (0/67)	8.3% (2/24)	**Fisher’s F test *p*-value = 0.07**

T0 = without any water treatment, T1 = after treatment the water with hydrogen peroxide and silver ions at initial concentration of 20 mg/L and after flushing water for 20 min/day for seven successive days, T2 = 15 days later; **bold** indicates statistically significant differences.

**Table 3:** Median microbiological load comparison between taps and showers.

Microorganism	Period	Tap–Median load (Range)	Shower–Median load (Range)	Test
Coliforms (CFU/100 mL)	T0	<1 (<1–35)	<1 (<1–19)	W = 784, *p*-value = 0.76
	T1	<1 (<1–60)	<1 (<1–10)	W = 827.5, *p*-value = 0.91
	T2	<1 (<1–3)	<1	W = 768, *p*-value = 0.23
*E. coli*	T1	<1 (<1–2)	<1	W = 1140, *p*-value = 0.30
(CFU/100 mL)				
Enterococci (CFU/100 mL)	T1	<1 (<1–3)	0 (0–2)	W = 847, *p*-value = 0.49
	T2	<1	<1 (<1–1)	**W = 737, *p*-value = 0.018**
*P. aeruginosa* (CFU/250 mL)	T0	<1 (<1–790)	2 (<1–3200)	W = 687, *p*-value = 0.26
	T1	<1 (<1–610)	<1(<1–26)	W = 823.5, *p*-value = 0.83
	T2	<1	<1 (<1–170)	W = 770.5, *p*-value = 0.099
*L. pneumophila* (CFU/L)	T0	1700 (<50–24,000)	325 (<50–22,000)	**W = 1091.5, *p*-value = 0.009**
	T1	<50 (<50–2300)	<50 (<50–2000)	W= 868, *p*-value = 0.5058
	T2	<50	<50 (<50–50)	**W = 737, *p*-value = 0.018**

T0 = without any water treatment, T1 = after treatment the water with hydrogen peroxide and silver ions at initial concentration of 20 mg/L and after flushing water for 20 min/day for seven successive days, T2 = 15 days later; **bold** indicates statistically significant differences.

**Table 4:** Poisson regression model of the *Legionella* load-preliminary and final models.

Indipendent Variables	**B**	(eβ-1) = RR (%)	*p*-Value
Intercept	7.06		<0.0001
Coliforms	7.28	1449.99	<0.0001
*E. coli*	−2.277	−1.00	<0.0001
Enterococci	−4.78	−0.99	<0.0001
*P. aeruginosa*	0.07	0.08	<0.0001

RR = Relative risk.

## Data Availability

Data are contained within the article.
